# Assessment of the effect of spacer material on gap and void formation in an endodontic temporary restoration using micro-computed tomography

**DOI:** 10.1038/s41598-023-31290-8

**Published:** 2023-03-16

**Authors:** Manal Alkadi, Fahda N. Algahtani, Reem Barakat, Rahaf Almohareb, Reem Alsaqat

**Affiliations:** 1 Dental Clinics Department, King Abdullah bin Abdulaziz University Hospital, Princess Nourah Bint Abdulrahman University, Box 84428, Riyadh, 11671 Saudi Arabia; 2 Division of Endodontics, Department of Clinical Dental Sciences, College of Dentistry, Princess Nourah Bint Abdulrahman University, Box 84428, Riyadh, 11671 Saudi Arabia

**Keywords:** Biomaterials, Biomaterials, Techniques and instrumentation

## Abstract

This study aimed to evaluate the effect of two spacer materials (cotton pellet and polytetrafluoroethylene [PTFE]) on gap and void formation in the Cavit restoration used for endodontic temporization. Thirty-four extracted human single-rooted premolars were matched and allocated into two groups (n = 17/group) according to the access cavity spacer (cotton pellet or PTFE). Standardized endodontic access cavities were prepared in all the teeth. Subsequently, the teeth were scanned by micro-computed tomography (micro-CT) to determine the volume of the access cavities. The teeth were then temporized by placing either cotton pellet or PTFE as spacer materials on the canal orifices, followed by the Cavit restoration. Following the temporization procedure, the teeth were subjected to a second micro-CT scan to determine the percentage volume of gaps and voids along the margins and within the Cavit restoration, respectively. Statistical analysis was performed using the Shapiro–Wilk and Wilcoxon signed-rank tests with a 5% significance level. The PTFE spacer was associated with significantly less gap formation between the Cavit restoration and the access cavity walls (*P* < 0.05) compared with the cotton pellet. No difference existed in void formation between the groups (*P* > 0.05). These findings indicate that the spacer material placed under the Cavit restoration can influence the quality of overlying restoration. PTFE was associated with less gap formation and, therefore, performed better than the cotton pellet as a spacer material beneath the Cavit restoration during endodontic treatment.

## Introduction

While there are several advancements in the endodontic field, including new concepts, materials, devices, and approaches; microbial eradication remains the main target of endodontic treatment. Therefore, efforts are directed toward achieving this target through proper chemical and mechanical treatment of the root canal space followed by hermetically sealing the disinfected space by utilizing biocompatible materials^1,2^. These treatment procedures might be performed in single or multiple visits. For several reasons, including time limitation, presence of pain, or placement of intracanal medicaments, the multiple-visit approach has been very common in endodontic practice^3,4^. In such cases, placing a temporary restoration is needed between the appointments. Temporary restorations are also commonly placed after the completion of endodontic treatment and before the placement of the definitive restoration^4^. Accordingly, temporization is considered an integral aspect of endodontic treatment and requires proper attention. Unsatisfactory temporary restorations ranked second amongst the factors associated with continuing pain after the initiation of endodontic treatment^5^. This should further emphasize the importance of providing an adequate seal of the disinfected root canals against the ingress of bacteria and organic materials from the oral cavity, whether between appointments or after completion of the endodontic therapy^4^.

As part of the temporization process and before placement of the provisional restoration, many clinicians prefer to place a spacer material on the floor of the endodontic access cavity, covering the canal orifices beneath the provisional restoration^3,6,7^. The main objectives of using these materials are to enhance the removal of the provisional restoration and prevent accidental blockage of the unfilled root canals by small pieces of the restorative materials^3,4,8–10^. Ideally, access spacers should enhance or at least not compromise the sealing ability of the provisional restoration^3^.

Currently, the cotton pellet is the most commonly used endodontic access spacer^6,7^. However, growing evidence has increasingly challenged its use^3,8–11^. The fibrous, organic, and hydrophilic nature of the cotton may promote bacterial growth and induce leakage^3,4,8^. In addition, the cotton fibers may become entrapped between the restorative material and the access cavity walls, leading to the formation of gaps or unfilled spaces in these areas, and subsequently promote further leakage^3,4,8–10^. Furthermore, the soft and porous nature of the cotton pellet may interfere with adequate compaction of the overlying temporary restoration, possibly leading to the formation of voids within the restorative material^3,8–10^. Recently, a different material has gained interest and been suggested as an alternative access spacer^3,8–10^. Polytetrafluoroethylene (PTFE) tape, commonly known as Teflon tape, is a polymer material that possesses several unique properties and has been used in different dental applications including endodontic access spacers^12^. It is non-biodegradable^13^, nonfibrous^8^, hydrophobic^9,13^ and autoclavable^3^. Additionally, it is firm when condensed; thus, it has been thought to allow for better compaction and increased thickness of the provisional restoration, compared with the soft cotton pellet. Moreover, it lacks the fibers that may interfere with the adequate adaptation of the restorative material to the walls of the access cavity^3,4,8–10^.

PTFE has been investigated as an alternative endodontic access spacer to the cotton pellet, both clinically^9,10,14^ and in laboratory studies^3,8^, and showed promising results. The PTFE spacer was consistently associated with lower levels of microbial contamination^3,8–10,14^. However, these studies are limited, and none has evaluated the effect of the spacer material on the marginal integrity or density of the provisional restoration. Therefore, this study aimed to evaluate the unfilled-space (gap and void) volume in the access cavities temporized using either cotton pellet or PTFE spacers beneath the Cavit restoration, using micro-computed tomography (micro-CT). The null hypotheses tested were the following:

1. There would be no difference in the percentage volume of gaps (gap%) at the Cavit restoration-tooth wall interface between teeth temporized with a cotton pellet spacer and those temporized with a PTFE spacer beneath the Cavit restoration.
2. There would be no difference in the percentage volume of voids (void%) within the Cavit restoration between teeth temporized with a cotton pellet spacer and those temporized with a PTFE spacer beneath the Cavit restoration.
3. There would be no difference in the total porosity (gap% + void%) of the Cavit restoration between teeth temporized with a cotton pellet spacer and those temporized with a PTFE spacer beneath the Cavit restoration.

## Materials and methods

The manuscript of this laboratory study has been written according to Preferred Reporting Items for Laboratory studies in Endodontology (PRILE) 2021 guidelines (Nagendrababu et al. 2021, https://doi.org/10.1111/iej.13542).

### Sample size calculation

The present study was registered and ethically approved by the institutional review board at Princess Nourah Bint Abdulrahman University, Riyadh, Saudi Arabia (reference no. 21-0263, IRB: H-01-R-059). Moreover, the collection of teeth and the experimental procedures were performed in accordance with the guidelines and regulations of the IRB at Princess Nourah Bint Abdulrahman University and the need for informed consent was waived by the IRB. The teeth were extracted for orthodontic or periodontal reasons and the patients provided informed consent for such treatments. The extracted teeth were kept in normal saline until their use in this study.

Given the lack of specific studies that evaluated gaps and voids in provisional restorations using micro-CT, a pilot study was conducted to estimate the sample size. The pilot study involved eight teeth that were matched into four pairs, as described later. G*Power 3.1 software for Mac was used (Henrich Heine-Universität, Düsseldorf, Germany). From the t-test family, a Wilcoxon signed-rank test was selected with an alpha-type error of 0.05 and power (1-B) of 0.95. The resultant effect size of 0.90 was obtained based on the results of the pilot study (voids% for cotton pellet and PTFE spacers, respectively, were 0.40 ± 0.15 and 0.27 ± 0.14). Based on these parameters, the required sample size was 17 specimens per group.

### Specimen selection and access cavity preparation

Thirty-four extracted human single-rooted maxillary and mandibular premolars with single canals were used. Only intact teeth that were caries-free with no resorptive defects or detectable cracks were included. The integrity of the crowns and the sigle root canal configuration were confirmed by the initial micro-CT scans. The teeth were arranged into 17 pairs. Pairing of teeth was carried out by direct visualization and using buccolingual and mesiodistal periapical radiographs and was based on the similarity of the size and dimensions of the crowns and pulp chambers. Appropriate teeth pairing was further confirmed by the initial micro-CT scan. The specimen selection and pairing processes were performed simultaneously by two endodontists.

To facilitate standardization of the access cavities, the cusps were reduced with a diamond bur until producing flat occlusal surfaces. The access cavities were then prepared on the occlusal surface with a high-speed tapered diamond bur (Kerr Dental, Orange, CA, USA), producing an oval access opening with the following dimensions: length of 3 mm, width of 2.5 mm and depth of 5.5 mm. For each tooth sample, a small access opening was prepared initially. Then, the following dimensions (width, length, depth) of the access cavity were measured using a UNC-15 periodontal probe (Hu-Friedy, Chicago, Illinois, USA). Subsequently, the access opening was adjusted gradually, and the dimensions were frequently checked using the periodontal probe until obtaining the desired measurements. The length of the access cavity referred to the longer diameter of its oval opening at the occlusal surface.The width of the access cavity referred to the shorter diameter of its oval opening at the occlusal surface.The depth of the access cavity referred to the distance from the occlusal surface to the most apical level of the cemento-enamel junction (CEJ). The 5.5 mm-depth of the access cavity was obtained by adjusting the level of the occlusal surface. The canal orifices were located and widened using a size #2 Gates Glidden (Dentsply Sirona). Using a rubber stopper, the Gates Glidden was inserted to a depth of 7.5 mm from the occlusal surface (2 mm beyond the predetermined 5.5 mm-depth of access cavity) so that its tip would reach the level just below the CEJ.

Apical patency was then obtained using #10 K-file (Dentsply Sirona) and the working length (WL) was determined to be 1 mm short of the point at which the #10 K‑file exited the apical foramen. To dissolve the pulp tissue, the canals were irrigated with a total volume of 5 mL 5.25% sodium hypochlorite (NaOCl; Clorox, the Clorox company, Dammam, Saudi Arabia) for 5 min using a 30-G Navitip (Ultradent Inc., South Jordan, UT, USA) inserted up to 2 mm short of the WL^15^. The irrigant was mechanically agitated for 30 s using #15 K-file (Dentsply Sirona) inserted to WL and moved manually up and down in short vertical strokes. Following that, the pulp chambers and canals were meticulously dried using a sequence of paper points sizes (ISO #50–15) (Meta Biomed, Korea) reaching to the WL.

### Preparation of PTFE pellets

A method described in a previous study was used^3^. A resin mould of a size-four cotton pellet was used to prepare the PTFE pellets (PTFE thread seal tape, ACE, Illinois, USA). PTFE tape was cut into 2-cm pieces, placed into the mould and adjusted to produce size-four pellets.

### Temporization procedure

The temporization procedure was conducted on the empty canals to simulate the clinical situation of temporization between endodontic appointments. After access cavity preparation and before the temporization procedure, the teeth underwent an initial micro-CT scan to determine the volume of the access cavity above the spacer material that would be placed later (occlusal 3 mm). The temporization procedure was then conducted on each pair of teeth, with one tooth receiving a cotton pellet (CP group, n = 17) and the other one receiving PTEF (PTFE group, n = 17) as access cavity spacers beneath the Cavit restoration. The teeth in each pair were randomly assigned to the study groups. The spacers were placed over the canal orifices with a standardized thickness of approximately 2 mm. Following the spacer placement, a periodontal probe was used to ensure a uniform and adequate remaining depth of 3.5 mm for the provisional restoration (Cavit). The Cavit (3 M ESPE, Seefeld, Germany) was placed over the spacer in two increments using a plastic instrument and condensed with a condenser until the access cavity was filled. To ensure a balanced and uniform temporization between the groups, the teeth were temporized in a sequence following the pairs in which they were matched. That is, after one tooth from the CP group was restored, the next one was from the PTFE group, which was the other tooth in the same pair. The access cavity preparation and spacer placement were performed by an endodontist, whereas the Cavit restoration was placed by a restorative dentist who was blinded to the aim of the study and the presence of different types of spacer in the access cavities before Cavit placement.

After the temporization procedure, the teeth were kept in wet gauze for 24 h to allow for the hygroscopic expansion of Cavit. Subsequently, they were subjected to a second micro-CT scan using the same parameters as the first. This was to evaluate the quality of the Cavit restoration by determining the volume percentage of empty space (gaps and voids) in the occlusal 3 mm of the access cavity. The 0.5 mm directly above the spacer was excluded from the analysis to avoid the possibility of including the radiolucent spacer as a false result of gaps or voids.

### Micro-CT evaluation and calculation of percentage volume of gaps and voids

All the teeth were scanned twice. The first scan was performed after the access cavity preparation to determine the volume of the access cavity (pre-temporization scan), and the second was performed after teeth temporization to determine the percentage volume of gaps and voids in the temporary restoration (post-temporization scan). To standardize sample positioning during the two scans, each tooth was embedded in resin and attached to a custom silicon mould. The teeth were scanned using a micro-CT device (SkyScan 1173; Bruker-microCT, Kontich, Belgium) according to the following parameters: 65 kV, 108 µA, pixel size of 12.7 µm, 360° rotation, rotation step of 0.3, an exposure time of 250 ms and 1-mm-thick aluminium filter. The obtained raw images were reconstructed using NRecon v.1.6.10 software (Bruker-microCT) with 25% beam hardening correction and ring artifact correction of 5, resulting in the generation of 1,115 transverse cross-sectional images per tooth. The data obtained from the post-temporization scan were coregistered with their respective pre-temporization data using Data viewer v.1.5.6.2 software (Bruker-microCT). The CTAn® v.1.20.8.0 software (Bruker-microCT) was used for the segmentation of the tooth structure, restorative material and empty spaces (gaps and voids) and quantitative analysis of the volumes (mm^3^) of access cavities, gaps and voids. Three-dimensional images were produced using the CTVol software (Bruker-microCT).

In this context, gaps were defined as the empty spaces located at the tooth–restoration interface, whereas voids referred to the empty spaces that were entirely contained within the restoration and did not communicate with the tooth walls^16^. The region of interest was selected as the occlusal 3 mm of the access cavity. The percentage volumes of gaps and voids were determined according to the following formula, which was used previously^16^:

$${\text{\% Gaps volume}} = \frac{{{\text{Gaps volume x }}100}}{{\text{Access cavity volume }}}$$

$${\text{\% Voids volume}} = \frac{{{\text{Voids volume x }}100}}{{\text{Access cavity volume }}}$$

In addition, the total porosity was evaluated. This referred to the sum of the percentage volumes of gaps (open pores) and voids (closed pores)^17^. The samples were coded before micro-CT imaging. All the scanning and analysis processes were performed in a dimly lit room by a micro-CT analyst who was blinded to the aim of the study and the allocation groups.

### Statistical analysis

Statistical analysis was conducted using IBM SPSS version 22.0 (Armonk, NY, USA). The normality of data distribution was evaluated by the Shapiro–Wilk test. Because the data were not normally distributed in at least one of the groups, the Wilcoxon signed-rank test was used to compare the different parameters between the CP and PTFE groups. A significance level of α = 0.05 was considered.

## Results

**Figure 1:**
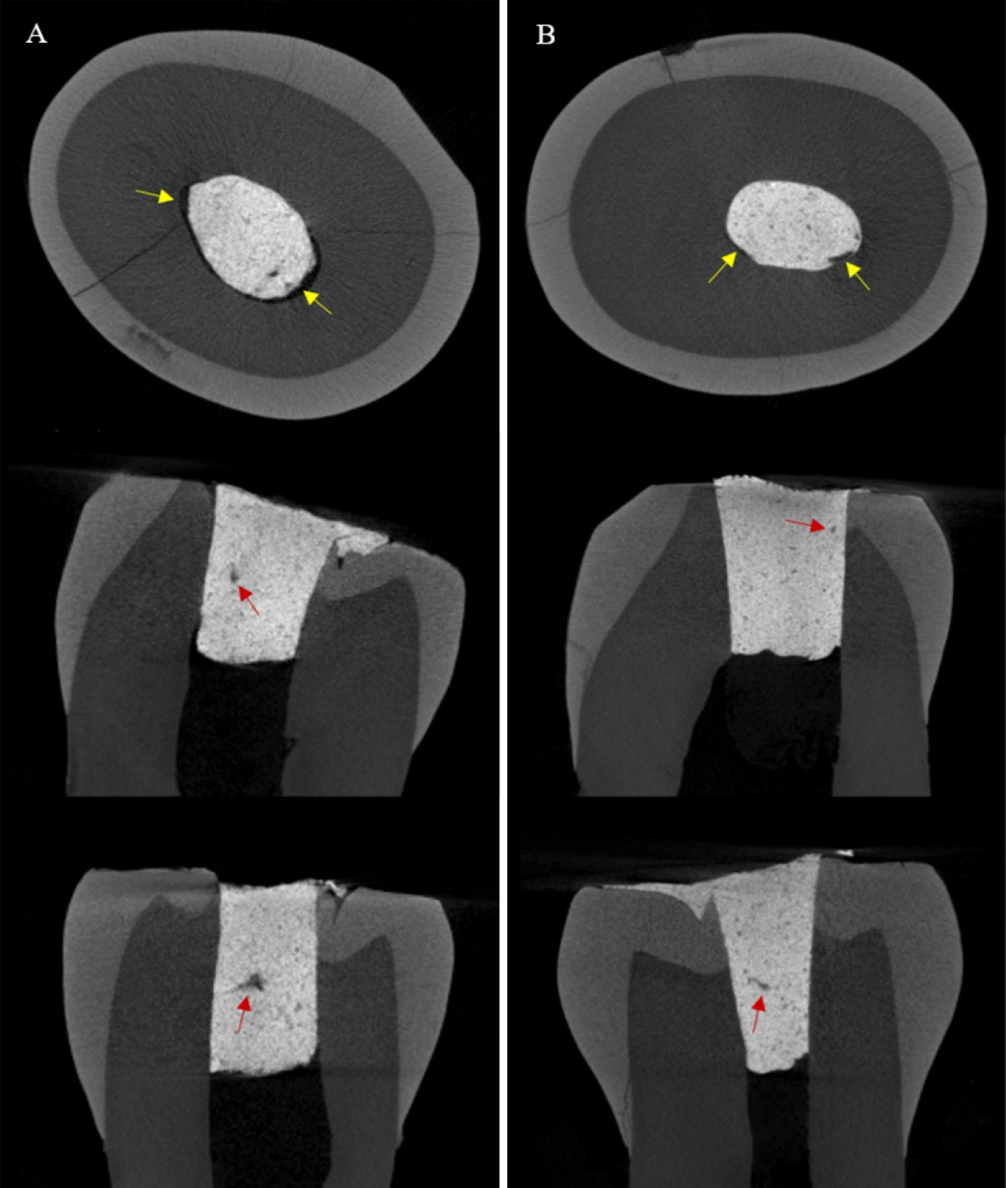
Representative micro-CT images (from top to bottom; axial, coronal, and sagittal views) of the two spacer groups: (**A**) cotton pellet, and (**B**) PTFE; showing the presence of gaps (yellow arrows) between the Cavit restoration and access cavity walls and voids (red arrows) within the Cavit restoration. The brightness of the original images was slightly adjusted to enhance visualization.

**Figure 2:**
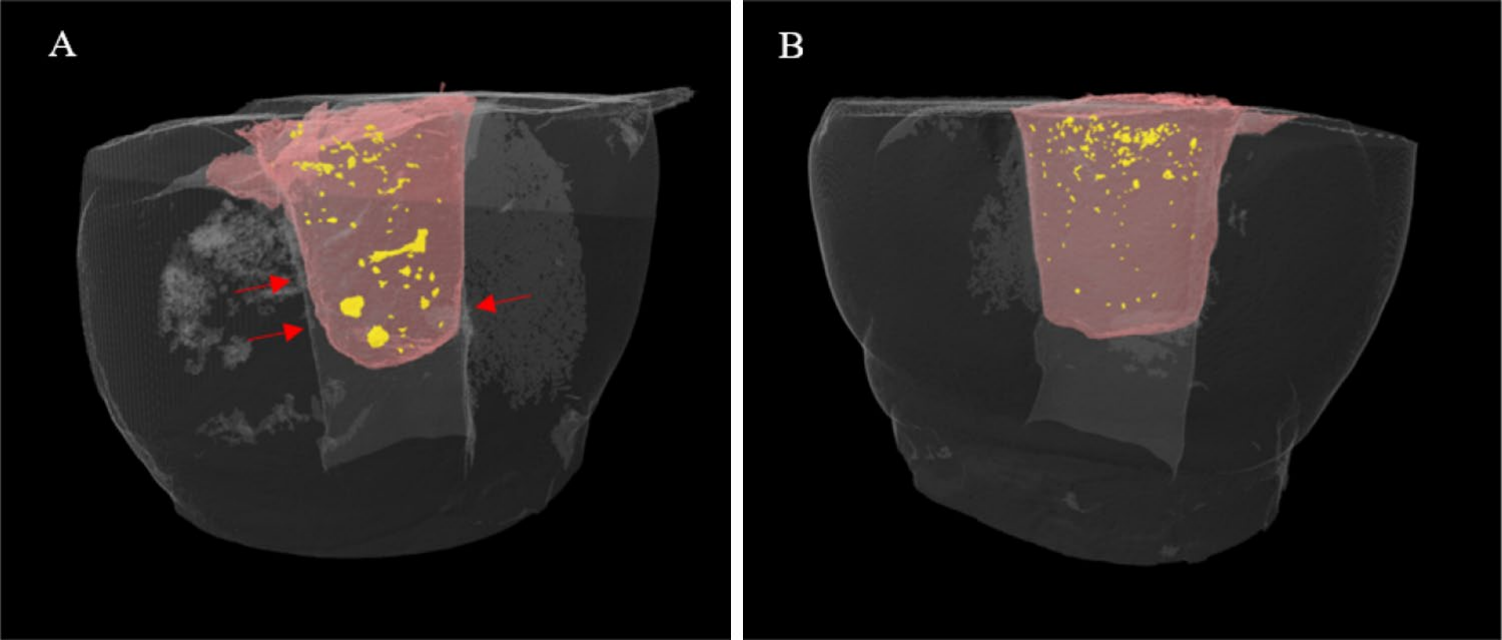
Representative 3D images showing the presence of voids (in yellow) in Cotton pellet (**A**) and PTFE (**B**) groups. It can be noted that with the PTFE spacer, the Cavit restoration had a flat surface over the spacer with a relatively uniform depth, compared with the cotton pellet that produced a convex restoration surface with marginal gaps (red arrows).

The access cavity volumes were similar in the CP and PTFE groups (*P* > 0.05), suggesting standardized access cavity preparation. Table 1 shows the percentage volumes of voids and gaps and total porosity for the two groups. The percentage volume of voids did not differ significantly between the groups (*P* = 0.76). However, significant differences existed in the percentage volume of gaps and total porosity between the groups, with higher volumes of gaps and total porosity in the CP group compared to the PTFE group (*P* = 0.008). Representative micro-CT and 3D images of both groups are shown in Figs. 1 and 2, respectively.

**Table 1:** Medians and interquartile ranges (Q1-Q3) of the percentage volumes of voids and gaps and total porosity for the cotton pellet and PTFE groups.

Group	Sample (N)	Voids% Median (Q1–Q3)	Gaps% Median (Q1–Q3)	Total porosity Median (Q1–Q3)
Cotton pellet	17	0.29 (0.18–0.35)^A^	1.72 (1.44–3.19)^A^	2.07 (1.54–3.55)^A^
PTFE	17	0.26 (0.19–0.35)^A^	0.97 (0.53–1.41)^B^	1.22 (0.93–1.61)^B^
*P*-value		0.757	0.008*	0.008*

Different uppercase letters in the same columon indicate significant differences between the groups.

*P < 0.05 by Wilcoxon signed-rank test. Q1, 25th percentile, Q3, 75th percentile.

## Discussion

Despite the extensive use of spacer materials in endodontic temporization, the effect of these materials on the overall quality of the provisional restoration has received only minor attention^7,11^. Therefore, the present study aimed to evaluate the effect of using either cotton pellet or PTFE as spacer materials on the marginal integrity and density of the overlying Cavit restoration. These two qualities of the Cavit restoration were respectively assessed by evaluating the gap formation between the restoration and the cavity wall, and the void formation within the restoration, using micro-CT. Previous studies investigated the effect of endodontic spacer material on the sealing quality of temporary restorations mainly by evaluating bacterial leakage, either in clinical^9,10,14^ or laboratory settings^3,8^. To our knowledge, no previous study has utilized micro-CT to evaluate the marginal adaptation or density of temporary fillings placed over different types of spacers.

Endodontic provisional restorations should prevent microbial contamination and maintain the canal space in the disinfected condition^4^. Existence of gaps between the cavity wall and the provisional restoration can affect its marginal integrity and lead to microbial leakage^16^. In the current study, teeth temporized with PTFE spacer showed less gap formation and total porosity compared to those with the cotton pellet, leading to the rejection of the first and third null hypotheses of this study. These findings may be explained by the presence of cotton pellet fibres that could interfere with the proper adaptation of the Cavit restoration to the access cavity walls and lead to gap formation. On the other hand, the PTFE spacer lack these fibres. Another possible reason may be related to the fluactuant and porous consistency of the cotton pellet, which may render the filling unstable during condensation and affect its adaptation both vertically to the underlying spacer surface and laterally to the cavity walls. In contrast, PTFE is more solid and allows for proper condensation of the provisional restoration^8^. It is worth noting, however, that gap generation is a complex phenomenon and cannot be related to a single factor. These factors may include cavity design, the technique of restoration placement, and condensation forces^18^.

Void formation is the other aspect of restoration quality that was evaluated in the current study. No difference was found in void formation between the cotton pellet and PTFE groups. In general, the percentage volumes of voids were relatively small for both groups, possibly leading to undetectable differences between the groups. It is generally believed that voids formation has less clinical significance than gap formation, particularly concerning microleakage. Voids are considered internal spaces that are enclosed within the restoration, while gaps are marginal spaces that can directly contribute to marginal microleakage^19^. Nevertheless, voids can be detrimental to the compressive strength of the Cavit restoration. Although voids may have a minor effect on microleakage, a negative correlation generally exists between voids/total porosity and the mechanical properties of the material, including compressive strength^20^. Cavit has a relatively low compressive strength, which could be further reduced by the presence of voids. A minimum thickness of 3.5 mm has been recommended to overcome this limitation and prevent leakage^21^, which was applied in the current study.

Overall, the present study demonstrated that PTFE performed better than the cotton pellet as a spacer material and resulted in improved quality of the overlying Cavit restoration with respect to gap formation and total porosity. Similarly, previous studies showed superior performance of the PTFE spacer compared to the cotton pellet^3,8–10,14^. However, significant methodological differences between the current study and previous ones prevent direct comparison.

Clinical studies have evaluated PTFE and cotton pellet spacers in patients receiving endodontic treatment and showed less microbial contamination of the PTFE spacers and their access cavities^9,10,14^. Olsson et al.^9^ investigated the PTFE spacer clinically in permanent molars. After pulp space debridment, the patients received either cotton pellet or PTFE spacers under the Cavit restoration. In the second appointment, the spacers were retrieved and evaluated for microbial growth by culturing on agar plates. Cotton pellet spacers were significantly more frequently contaminated (15/24, 63%) compared with PTFE spacers (2/24, 8%)^9^.

Moreover, two clinical studies were conducted on primary molars^10,14^. Following the pulpectomy procedure, the patients received either cotton pellet or PTFE spacers beneath the Cavit restoration. After seven days, the spacers were retrieved, and samples were obtained from the access cavities for culture. Cotton pellet spacers and their associated access cavities showed significantly higher microbial colony counts compared with PTFE spacers.

Laboratory investigations have also shown similar findings. Paranjpe et al.^8^ prepared access cavities in extracted human molars and restored them with either cotton pellet or PTFE spacers followed by a Cavit restoration. The coronal portions of the teeth were immersed in a bacterial broth for seven days, after which, the spacers were retrieved, placed on agar plates, and observed for bacterial growth. Nine out of the 10 cotton spacers were positive for bacterial growth compared with only one of the 10 PTFE spacers. Additionally, an ex vivo study that utilized molecular analysis using real-time polymerase chain reaction reported lower counts of leaked bacterial cells through the root canals in teeth restored with PTFE spacer, compared with the cotton pellet^3^.

The current study utilized micro-CT scanning as the method of evaluation of two types of restoration porosity (gaps and voids). Other methods that can be used for the same purpose include scanning electron microscopy and conventional radiography^19^. The use of micro-CT has several advantages over the other methods. It is non-destructive and accurate and allows 3D visualization of the specimens^16^. It can be utilized for qualitative and quantitative analysis and has been used previously to evaluate the marginal adaptation of dental restorations^18,22^.

Although complete elimination of sources of bias is difficult to ever guarantee, efforts were made in the current study to reduce biases. For example, the spacer material and the Cavit restoration were placed by two different operators. An endodontist placed the spacer materials and then measured the depth of the access cavity to ensure an adequate depth of 3.5 mm for the Cavit restoration. Subsequently, a restorative dentist was asked to place the Cavit restorations. The latter was not aware of the aim of the study or the presence of different types of spacers in the cavity before Cavit placement. Furthermore, the teeth were matched into pairs. The benefits of the pairing process in minimizing anatomical bias and increasing the internal validity of the study are well known^16^. Additionally, the teeth were restored in pairs regarding the sequence of restoration placement. That was in an attempt to ensure uniform condensation forces between the groups, which may fluctuate during the procedure or decrease over time due to operator fatigue. The size of the spacer material was standardized for both groups and was equal to that of a size-four cotton pellet by utilizing a resin mould designed specifically for this purpose. The teeth were kept in wet gauze for 24 h to simulate the moist condition of the oral environment and allow for hygroscopic expansion of Cavit due to water sorption, since this property may affect the formation of gaps and voids. Cavit was chosen as the provisional material to be evaluated since it has been one of the most commonly used provisional restorations in endodontics^6,7^. It is easy to apply and remove and generally shows adequate qualities as an endodontic provisional restoration^4^.

The results of the present study should be interpreted carefully. Although it has been reported that neither the dimensional stability nor the sealing ability of Cavit was affected by temperature fluctuations^23,24^, other conditions of the oral environment such as masticatory forces and the combination of these forces with temperature changes were not simulated. Additionally, the analysis was limited to a class I access cavity design and a single type of teeth and restorative materials. Therefore, the applicability of the findings to other tooth types, access cavity designs, and provisional restorations may not be guaranteed.

In line with the findings of previous studies^3,8–10,14^, the present study indicates that the widely used cotton pellet spacer may not be the best option for this purpose and the PTFE spacer is a potential alternative. Since the spacer material has been shown to affect different qualities of the temporary restoration^3,8–10^, future studies need to devote more focus to investigating this aspect of endodontic temporization. Furthermore, it is recommended to investigate other materials as spacers and in combination with different temporary restorative materials. New studies may include clinical investigations and laboratory studies that incorporate thermo-mechanical cycling with different tooth types and access cavity designs.

## Conclusion

The findings of this ex vivo study demonstrated that the type of spacer material placed beneath the Cavit restoration could influence the quality of temporization. Specifically, the PTFE spacer was associated with a lower potential for gap formation between the Cavit restoration and the access cavity walls. It thus performed better compared with the more commonly used cotton pellet and may be considered clinically as a potential alternative spacer.

## Supplementary Information

Supplementary Information.

## Data Availability

The data that support the findings of this study are available from the corresponding author upon reasonable request.
